# Blocking the Reflection: Milestones and Hurdles for Digital Twins in Mental Health

**DOI:** 10.1055/a-2816-2869

**Published:** 2026-03-19

**Authors:** Falk Gerrik Verhees, Isabella Catharina Wiest, Jakob Nikolas Kather, Joseph Kambeitz, Pavol Mikolas

**Affiliations:** 1 59988 Department of Psychiatry and Psychotherapy, Faculty of Medicine Carl Gustav Carus, TUD Dresden University of Technology, Dresden, Germany; 2 99045 Department of Medicine II, Medical Faculty Mannheim, Heidelberg University, Mannheim, Germany; 3 9169 Else Kröner Fresenius Center (EKFZ) for Digital Health, Faculty of Medicine and University Hospital Carl Gustav Carus, TUD Dresden University of Technology, Dresden, Germany; 4 National Center for Tumor Diseases (NCT), Heidelberg University Hospital, Heidelberg, Germany; 5 Department of Medical Oncology, Heidelberg University Hospital, Heidelberg, Germany; 6 Department of Psychiatry and Psychotherapy, University Hospital Cologne, University of Cologne, Cologne, Germany; 7 28302 Max Planck Institute for Metabolism Research, Cologne, Germany; 8 39065 Department of Psychiatry and Psychotherapy, Jena University Hospital, Jena, Germany

**Keywords:** artificial intelligence, digital twins, precision psychiatry, LLM

## Abstract

Artificial intelligence in mental health has emerged as a potent tool to foster precision psychiatry, for example, by stratifying patient populations. A potential step forward would be mental health digital twins—the independent in-silico reconstruction of an individual person within their functional social and environmental systems that continuously incorporate all known and available subject parameters to predict patient trajectories including the outcomes of interventions. Generative artificial intelligence in the form of large language models demonstrated the ability to mimic human responses and integrate diverse sources of information that may foster the development of digital twins. We give a brief historical perspective on concepts and milestones of artificial intelligence in mental health and outline the current state of clinical decision support systems, monitoring and therapy applications based on artificial intelligence. We describe their integration in large behavioral models as a recently met precondition for digital twins and contrast this development with the magnificent hurdles that remain to truly realize clinical benefits of digital twins, from data quality and regulatory compliance to user engagement and public trust, for some of which we propose mitigation strategies here.

## Introduction

**Fig. 1:**
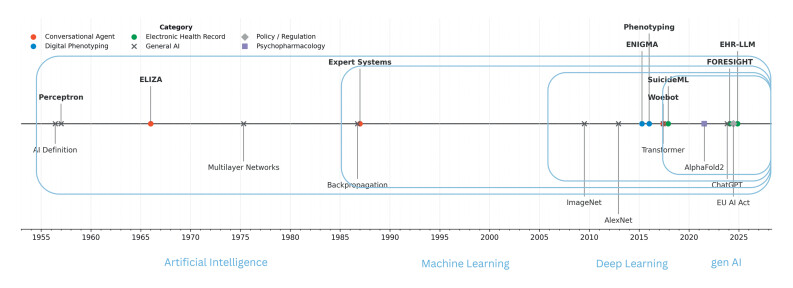
A brief history of AI, with special regard to mental health. Light blue ellipsoids represent the conceptual familial hierarchy of genAI within the areas of deep learning, machine learning and AI in general. Above timeline: selected milestones related to mental health. The perceptron model as a brain-inspired basis for artificial neural networks, 43​ ELIZA. 17​ Digital phenotyping in the early 2010s, 34​ big-data consortia like ENIGMA as a basis for AI in psychiatry, 44​ suicide prediction, 36​ Woebot, 37​ Foresight 45​ and other Electronic Health Record-based predictions. 40​ Below timeline: selected milestones for AI in general. Thematically grouped, see legend. AI, artificial intelligence; genAI, generative AI.

**Fig. 2:**
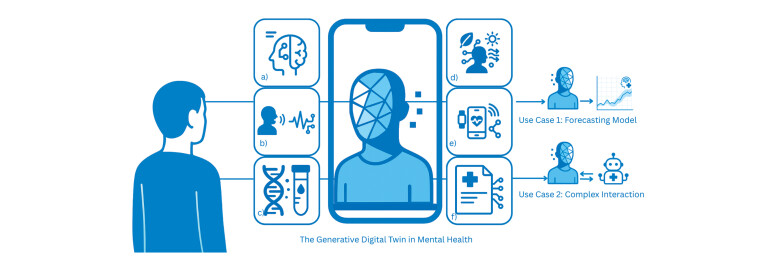
The generative DT in mental health. A coordinating agentic model incorporates various input streams to accurately capture behavioral and molecular processes. ( **a** )–( **f** ): DTs input modalities. ( **a** ) Neuroimaging data and ( **f** ) MRI. ( **b** ) Speech content and audio. ( **c** ) Genetic and biochemical markers, e.g., genome-wide associations or inflammatory markers. ( **d** ) Environmental variables, e.g. heat waves. ( **e** ) Digital phenotyping, e.g., passive sensing of movement or smartphone use patterns. ( **f** ) Electronic health records and primary care data for research use. Use cases 1 and 2 to illustrate the potential application of DTs, such as (1) as a prognostic model for symptom trajectory prediction or (2) as a validation agent for therapeutic interventions equally enabled by genAI that would be impossible to stress-test without DTs or other bespoke agents. Designed with Canva. AI, artificial intelligence; DT, digital twin; genAI, generative AI.

Precision psychiatry aims to match the right patient with the right diagnosis and the right treatment at the right time. 1​ ​2​ What sounds trivial is in fact a tremendous challenge: patients with bipolar disorder or psychosis oftentimes only receive the correct diagnosis and subsequent treatment after a long time. 3​ ​4​ Moreover, first-line treatments only lead to remission in up to 28% of cases with major depressive disorder (MDD), 5​ ​6​ with only 15% of patients experiencing a significant anti-depressive effect compared to the stricter control condition of a placebo in their first treatment attempt. 7​ The same observation holds true for psychotherapeutic 8​ or life style interventions. 9​ There is an urgent need for better biomarkers to identify the appropriate therapy strategy for each patient and reduce the need for trial-and-error. 2​ Beyond neurobiology-informed approaches, artificial intelligence (AI) appears to be a vital component for the better stratification of patient cohorts by supplementing theory-driven mechanistic discovery with the data-driven identification of relevant influences on mental health. 10​ 11​ ​12​ An understanding of both the history and methodological foundations of AI ( Fig. 1 ) is necessary to understand possibilities and limitations of the technologies, in psychiatry and beyond. A promising concept for precision medicine is arguably the digital twin (DT), a real time *in-silico* model of a person that would allow for timely diagnoses and treatment decisions by extrapolating from the DT back to the human original ( Fig. 2 ), even though formidable hurdles to its realization remain ( Table 1 ).

**Table:** **Table 1** Pre-requisites for safe and equitable DT deployment in mental health

Hurdles	Description	Mitigation strategy
Data integration and interoperability	Combining multimodal data (electronic health records, ecological momentary assessments, wearables, voice, and imaging) into a standardized, longitudinal representation of the patient.	Mental health data are fragmented and non-standardized; interoperability via FHIR/OMOP/LOINC is essential for scalability and real-world deployment. 98​ Without harmonized input streams, no twin can update or generalize. 48​
Longitudinal model validity and updating (VVUQ)	Models must remain valid over time, detect drift, and transparently manage uncertainty.	Verification, validation, and uncertainty Quantification (VVUQ) are indispensable for trustworthy DTs. 99​ ​100​ Mental health data are noisy and time-dependent, so adaptive calibration is a clinical safety requirement.
Clinical actionability	Translating predictions into interpretable, context-aware treatment recommendations (e.g., relapse alerts, medication adjustments, and live prompts).	Many prototypes stop at prediction accuracy; few connect to real interventions. Clinically actionable DTs embed human-in-the-loop decision logic to improve outcomes. 101​ ​102​
Evidence from prospective validation and trials	Demonstrating the real-world benefit via pragmatic or adaptive trials (e.g., SMART and micro-randomized).	Most mental-health AI lacks prospective validation; 42​ regulatory approval and adoption depend on outcome improvement. 99​
Regulatory and ethical compliance	DTs offering treatment recommendations are classified as high-risk AI-based SaMD, requiring human oversight, transparency, and post-market surveillance.	Compliance frameworks now converge on explainability, continuous monitoring, and bias auditing. These are non-optional for clinical deployment. 103​ 104​ ​105​
Explainability and clinician/patient trust	Generating human-understandable rationale for outputs and visualizing uncertainty and longitudinal changes.	Mental health clinicians must justify actions; opaque “black-box” systems hinder adoption and raise liability concerns. Interpretability drives trust and safe workflow integration. 106​ 107​ ​108​
Data privacy, security, and governance	Protecting highly sensitive personal data (voice, location, text) via federated learning, consent management, and robust audit trails.	Mental health data are among the most sensitive categories; legal and ethical constraints 103​ 109​​ ​110​ make secure architectures a prerequisite.

The intellectual roots of AI and thus the basis for the DT concept trace back to ideas of mechanizing reasoning, but the formal field of AI was born in 1956 at the Dartmouth Summer Research Project, where John McCarthy and colleagues proposed that “every aspect of learning or any other feature of intelligence can in principle be so precisely described that a machine can simulate it.” 13​ ​14​ Early systems in the late 1950s and 1960s emphasized symbolic reasoning and rule-based AI, such as expert systems. 15​ The 1966 chatbot ELIZA by Joseph Weizenbaum represented one of the first experiments at natural–language interaction, famously mimicking a Rogerian psychotherapist via pattern matching, 16​ ​17​ which heralded early interest in psychological applications *.* In subsequent decades, expectations outpaced progress. The field experienced cycles of “AI winters” in the 1970s and again in the late 1980s when funding and enthusiasm contracted, due to computational limits and unmet promises. With the resurgence of machine learning (especially statistical methods) in the 1990s and the rise of support vector machines, random forests, and early neural networks, AI began shifting from hand-coded rules to data-driven inference. 16​ The growing popularity of deep learning since 2012 relied mainly on specialized neural networks together with parallelized scaling of computation on dedicated graphics processing units instead of central processing units. Recurrent or convolutional neural networks behind such projects like AlexNet 18​ accelerated capabilities in pattern recognition and representation learning. More recently, transformer based architectures 19​ and the subsequent development of generative pre-trained transformers 20​ led to renewed public attention to AI. During the 2010s and into the 2020s, generative models (e.g. generative adversarial networks) and large foundation models (e.g. large language models [LLMs]) have redefined what is possible in prediction, synthesis, and representation learning. 21​ ​22​ LLMs are “foundation models,” i.e. pre-trained neural networks that learn representations from large corpora of the text available on the internet, including medical and psychological literature studies. With little “prompting,” i.e. instructions in the natural language, they are apt in generalizing capabilities across domains for which they have no specific training. 23​

The field of natural language processing (NLP) was in fact upended by the advent of LLMs, 24​ with considerable consequences for mental health research and, potentially, practice. Here, efforts to apply AI began early but remained largely exploratory: the aforementioned ELIZA experiment sparked ideas of therapeutic bots. This concept was followed by rule-based psychiatric expert systems for diagnostic reasoning in psychotic disorders, 25​ and diagnostic algorithms across mental disorders. 26​ Only decades later did AI models emerge that classify psychiatric symptoms, predict treatment outcomes, or assist in digital interventions 27​ directly from patient-generated data and bio signals. Acoustic speech biomarkers processed with machine learning algorithms support diagnosis and group stratification in MDD, schizophrenia and Alzheimer’s disease. 28​ 29​ ​30​ Public social media posts are highly accurate in predicting depressive symptoms using both supervised 31​ and unsupervised learning strategies. 32​ Smartphone-based “digital phenotyping” links passively sensed behavior to mental states and relapse risks. 33​ ​34​ At the level of health systems, large-scale EHR-based models have been developed to predict suicide attempts and deaths in routine care. 35​ ​36​ Fully automated cognitive behavioral therapy-based conversational agents such as Woebot have shown symptom reduction in randomized trials for depression and anxiety. 37​ Multi-site deep learning models trained on neuroimaging data alone 38​ and multi-modal data streams 10​ support diagnosis in schizophrenia and related disorders. More broadly, systematic evidence syntheses describe deep learning in psychiatric neuroimaging as the dominant methodology (Quaak et al., 2021). In parallel, the most recent generation of LLM-based systems is now being evaluated on mental-health–relevant NLP tasks such as stress, depression, and suicidality detection from the publicly available text 39​ and real world electronic health records (EHR). 40​ Despite important early successes, the integration of AI into clinical psychiatry remains nascent. Challenges of data heterogeneity, bias, interpretability, domain shift, regulatory constraints, and the need for human–AI collaboration continue to obstruct the path forward. 41​ Still, most models in psychiatry remain unvalidated research projects or, at best, at the retrospective validation stage. Relatively few have been externally validated in real-world clinical settings, 42​ and to our knowledge, none evaluated prospectively.

While generative AI (genAI) will likely transform all aspects of mental health care, including training and research, 46​ ​47​ we will primarily focus here on novel clinical applications of genAI and the possibility of individualized diagnosis and treatment options enabled by DTs, 48​ the construction of an *in-silico* representation of single patients. LLMs have been demonstrated to encode more clinical knowledge including the domain of mental health than other prior AI tools before 49​ and will thus be the focus of this review.

GenAI’s impact on neuro-psychopharmacology is noted mainly through drug-discovery pipelines: AlphaFold-driven protein-structure prediction 50​ and small-molecule design platforms have already identified novel receptor agonists for potential use in schizophrenia. 51​ Some of the compounds discovered using AI are currently moving into phase III clinical trials, 52​ ​53​ which is discussed in another contribution to this issue. Yet, in psychiatric practice, these advances remain further upstream compared with the more immediate clinical touchpoints offered by therapybots and DTs.

## Clinical applications

While many LLM-powered applications aspire to be used in health care, 54​ only one system (Prof. Valmed) for individualized clinical decision support (CDS) is currently (January 2026) classified as a medical device in the European union. 55​ The high usefulness demonstrated in clinical proof-of-concept studies on agentic reasoning in oncological tumor board decisions 56​ makes it likely though that the early trailblazers for clinical deployment will be joined by further products specifically tailored to mental health. They will influence clinical decision making, continuous monitoring, therapeutical interventions and will eventually contribute to the idea of DT, as outlined below.

### Clinical decision support

Although LLMs are not currently incorporated in medical devices for mental health, AI already plays an increasing role: rule-based chatbots that deliver elements of cognitive behavioral therapy like Moodgym or Woebot have been increasingly flanked by probabilistic systems for CDS. One such product (limbic.ai) increases accessibility to mental health care in the UK and the USA with a self-referral chatbot tool, 57​ much like a digital front door. While not yet in clinical use, a recent treatment response prediction model supports the choice of the right pharmacotherapy for the right patient out of 10 antidepressants in MDD across 9,042 patients with promising accuracy (area under the curve = 0.65). 58​ The decision when to supplement pharmacotherapy with psychotherapy could also be meaningfully supported by AI recommendations, which led to a significant reduction in Hamilton depression scale (HAMD) outcome scores by an average of 4.6 in a sample of 248 patients in retrospective analysis. 59​ Promising as they may be, the translation of AI-based CDS into clinical routine crucially hinges on user trust in the systems’ reliability: if caregivers do not trust AI decision-support tools and ignore their recommendations, they cannot have an impact on patient care. In one study, physicians mainly stuck to their own initial hypotheses for plausible differential diagnoses in case vignettes, even though the LLM-based CDS offered superior advice. 60​ Clinicians that had access to CDS based recommendations scored only 76% of cases correctly, compared to 92% of cases for the CDS alone.

### Monitoring

Patients who consent to contribute data from the devices in their pockets, such as smartphones and wearables, may profit from individualized monitoring and trajectory prediction. For example, in bipolar disorder, passive sensing of activity markers and changes in their autocorrelation allow for modest extrapolations about the course of the disease potentially anticipating (hypo)manic episodes in a frequently sampled cohort of 29 patients throughout 12 months. 61​ For the MDD, sensor data captured throughout 10 months for 183 patients allowed predictions about depression severity up to 3 weeks in advance with high accuracy (R2 ≥ 80%). 62​ A separate study shows additional value for capturing voice and speech data, 63​ based on NLP. In this domain of AI, LLMs have been shown to capture Beck Depression Inventory-II scores from real life chat protocols with high accuracy, 64​ opening another route for digital phenotyping for example in interactions on social media, that have been demonstrated to impact mental wellbeing, especially in vulnerable groups like children and adolescents, 65​ and are an increasingly relevant sphere of everyday life.

### Intervention

Well-timed interventions could built upon such an abundance of available monitoring data and can be administered by AI systems: the chatbot “Therabot” recently demonstrated effectiveness in reducing symptoms of anxiety and depression while maintaining a measure of therapeutic alliance with a mainly LLM-based digital intervention, 66​ in contrast to earlier chatbots as discussed above. Even though the study’s methodology can be criticized, for example the intervention group was compared to a waiting list control group, 67​ these results promise high value to patients. Further evidence points to a high degree of perceived empathy for several LLM chatbots in medical settings 68​ 69​ ​70​ that support the claim of therapeutic effects based on strong alliance.

The eventual and comprehensive combination of such AI based approaches in monitoring, clinical decision making and therapy might be an integrative system that allows for a virtual mirror image of a patient, a DT.

### Digital twins

DTs in mental health describe the independent *in-silico* reconstruction of an individual person within their functional social and environmental systems. In distinction with the predictive models described above, a DT will continuously incorporate all (or at least all known and available) subject parameters. 71​ A DT might use for example predictive models for extrapolation of future mental states and behaviour, but allows for feedback loops and testing parameter changes, for example by lifestyle modifications or (pharmaco-)therapeutic interventions. The concept of DTs originated from physical systems that could be built to scale in an artificial environment and allowed mirroring from afar. 72​ The concept was pioneered during the Apollo 13 mission in April 1970, the crew lost air purification and was instructed by ground-based engineers to improvise a replacement with the available equipment, mirrored on Earth. This success led to the crew’s safe return and is considered the birth hour of DTs. 73​

Ideally, a DT would (1) integrate all available data about an individual patient and would (2) use these data to predict outcomes, such as behavior or therapy response, referring back to a reliable model of behavioral or neurobiological mechanisms. While these preconditions are by no means meant to be exhaustive, they are certainly necessary to integrate DTs meaningfully into clinical practice. On the modelling of complex human behavior, recent progress demonstrates the ability of fine-tuned open-weight models, which are more transparent and controllable than their more prominent, proprietary counterparts, 74​ in correctly predicting behavioral responses. The Centaur model (based on Meta’s Llama-3.1-70B-model) was (in addition to its pre-training on a general test-corpus representative of the whole of the internet) exposed to the results of 160 psychological experiments. 75​

Recently, LLMs have been adopted to the task of predicting next health events in a patient trajectory, just like the next token in natural language: “Foresight” “DELPHI-2M,” a model based on an extended transformer architecture was able to predict long term disease trajectories trained on a comparatively small data set of 400,000 EHR files, performing best for mental disorders 76​ and outperforming standard risk prediction methods for dementia. In parallel, DT-GPT, a model trained on data from EHRs beat established risk prediction algorithms for the onset of Alzheimer’s disease by a relative improvement of 1.8%, although not statistically significant, in contrast to improved predictive performances of 3.4% and 1.3%, respectively, for non-small cell lung cancer and intensive care unit patients. 77​ Presumably, these disease and behavior models would have to be integrated into a multi-modal fusion of patient inputs (see Fig. 2 ) to capture the full trajectory of their mental health in a clinically useful DT. Generative AI offers a practical path for such integration: “generative agents” that can be designed to act as interactive simulacra of human behavior have already been realized in sandboxed environments and show believable, emergent social behaviors that can be instrumented and logged for evaluation. 78​ Emerging work proposes adapting these agentic simulations to mental health contexts so that socio-environmental stressors, coping routines, and care pathways can be explored safely before patient-facing deployment. 79​ In an agentic system, specialized models can collaborate, for example, a coordinator (“ego”) routes tasks to sub-agents for perception, memory, forecasting, and counterfactual reasoning. Such an architecture has already been validated in oncology for complex multimodal decision support. 56​ The principle of aggregating clinical data and executing the DT model with a suitable agent has been demonstrated recently for metastatic uterine carcinosarcoma, a rare gynecological tumor: an LLM based CDS recommended novel treatment strategies based on general demographic criteria and few selected biomarkers. 80​ While such a system would have to contend with the absence of biological biomarkers in mental health, a wealth of digital phenotyping data might fill the data gap: the twin could fuse longitudinal biomarkers from psychometrics, MRI, speech and language, and routine EHR streams with behavioral agents.

Prognostic models for disease-course prediction exist across serious mental illness: multimodal pipelines combining clinical, neurocognitive, MRI and polygenic risk scores predict psychosis transition with external validation; 10​ disorder-specific work shows an estimated risk for bipolar disorder from sMRI features, 81​ ​82​ and EHR-based progression to schizophrenia vs. bipolar disorder, 83​ while NLP augments these predictors: psychosis-risk calculators improve when enriched with clinically salient features mined from notes, 84​ and generative LLMs trained on whole timelines can model individual patient trajectories across specialties. 45​ The twin should also incorporate a rich picture of social determinants extracted from the unstructured medical text, like employment, housing, transport, and social support, where modern LLMs now achieve solid macro-F1 performance. 85​ For transdiagnostic psychopathology, end-to-end information extraction pipelines tailored to clinicians demonstrate feasibility for extracting features from psychiatric EHRs. 40​ 86​​ ​87​

Generative models also enable interpretable neurobiological mapping: diffusion models can convert a patient’s MRI into a personalized “healthy” counterfactual and derive voxel-level disease effect maps, an intuitive bridge between model outputs and clinical reasoning. 88​ ​89​ Finally, agent-based “AI hospital” simulators provide safe testbeds where multi-agent LLM systems (doctors, patients, and examiners) practice history-taking, test ordering and diagnostic reasoning, supporting rapid iteration on digital-twin workflows before touching real patients, 90​ ​91​ where models can be influenced by texts deemed traumatizing to exhibit anxious behavior. 92​ ​93​

Taken together, an agent-orchestrated DT incorporates (a) the modalities shown in Fig. 1 ; produces (b) at least calibrated risk, counterfactual explanation, and an action recommendation with rationales as an output (compare “use cases” in Fig. 2 ); and is (c) validated first in AI-hospital simulations, before running in shadow mode, with a subsequent prospective trial, addressing some of the challenges outlined below.

#### Current limitations and frontiers

All models are wrong, but some are useful, goes the statistical saying. Yet, both imperfect and—hypothetical—perfect DTs would confront us with challenges and limitations that stretch from basic mechanistic understanding of the human body and mind to the underlying models’ limitations in architectures and training data, and finally leading to relevant regulatory and ethical considerations. Primarily, the current understanding of mental health and its molecular underpinnings are more incomplete than would be required for a DT. The analogy to the original approach in the realm of engineering, as outlined in roadmaps for precision psychiatry, 2​ thus remains far from perfect. Very few deep learning models that could be incorporated into a DT are externally validated and, more critical, prospectively tested and implemented in actual clinical care, 42​ severely limiting the tooling a DT could reliably incorporate. The generative models themselves that could form the core of an agentic DT as described above are currently lacking in essential meta cognition, that is being unable to recognize and handle uncertainty in their outputs: LLMs “do not know what they do not know.” 94​ This shortcoming is also discussed as a reason for stark divergences between human and LLM performances on psychometric tests, which turn out to be of little use for these systems 95​ ​96​ and point to them being restricted in truly modelling a human counterpart as DT. They are also prone to indulge delusional or harmful content leading to output deterioration. 97​ Further technical (blue), clinical (green) and regulatory (orange) hurdles are outlined in Table 1 .

A clinically actionable mental health DT will only emerge when multimodal data streams are interoperable, models are dynamically validated and explainable, and their outputs are embedded in regulated, trial-tested decision workflows that clinicians as well as patients trust ( Table 1 ). This will include the potential for causality analyses that should be part of the explainability. Here, engagement with regulatory authorities will be a necessary pre-requisite to enable safe and equitable deployment and safeguards clinical AI applications in general and DT use, specifically. As the input and output space of such tools will be vastly greater than that of previous, deterministic products in healthcare, clinician input will be required to guide medical device certification along the way. 105​ Benchmarking efforts are becoming increasingly more sophisticated but still struggle to capture clinically meaningful, complex multiturn interactions between users and genAI, like MedHELM. 111​ Corporations, such as OpenAI, maker of ChatGPT, are also actively preparing their entry into the medical domain and try to implement industry-led self-evaluation with HealthBench. 112​ Tellingly, both MedHELM by an academic consortium and HealthBench devote less than 10% of their clinical cases to mental health and are thus sub-optimal for evaluating therapeutic interventions reliant on AI-enabled chatbots.

## Outlook

While the challenges to the implementation of DTs in mental health remain formidable, the application of their less sophisticated versions outside the academic realm demonstrates that individual behavior—at least for individuals with extensive written documentation—is feasible: “SCOTUS-bot” correctly predicted outcomes of the decisions of the 9 US-American Supreme Court judges in six out of seven cases, if not consistently down to the level of the individual vote. 113​ Likewise, complex negotiation behavior in high stakes situations has been modelled as a training tool. 114​ Both use cases show that in principle, LLMs hold sufficient knowledge about human decision making and behavioral processes to accurately model them in certain scenarios. As a starting point, judges’ dogmatic legal opinions can be replaced with patient belief systems and negotiations with automatic thoughts and schemata, and the basis for a DT in mental health is emerging. As outlined above, the models that underpin our conception of the DT in mental health are already deployed directly to health care professionals (Prof. Valmed and limbic) and patients alike (Woebot, Therabot, and limbic) and are firmly rooted in clinical practice. Thus, even if superhuman “artificial general (medical) intelligence” is not imminent, genAI is already extremely capable and useful “as I” and if no further progress in model capabilities could be achieved. The productive applications supporting mental health care today make the progress to digital twinning more likely tomorrow.

As long as we do not understand the underlying mechanistic principles of mental health well enough to specify which datasets must be integrated, we will remain severely limited in building a faithful and actionable DT. Additionally, even if these data requirements were to be known, the tools needed for integration (such as multimodal AI models, LLMs, and autonomous agents) are still imperfect, especially in handling heterogeneous clinical data reliably. Many of the potentially required data sources are either inaccessible, siloed, or not collected yet at the necessary granularity. Finally, the regulatory landscape surrounding the implementation, validation, and clinical deployment of DTs remains underdefined, making safe and responsible use difficult. In this sense, one can clearly state that we are not ready yet for the full clinical realization of patient-level DT, neither technically, nor infrastructurally, nor regulatorily. But conceptually, DTs are a highly promising tool for matching the right patient with the right diagnosis and the right treatment at the right time.

## Declaration of GenAI use

During the writing process of this paper, the author(s) used ChatGPT 5.1 Thinking in order to supplement the literature search and improve readability of the initial draft. Canva was used to create or adapt some of the icons in Fig. 2 . The author(s) reviewed and edited the text and visual output and take(s) full responsibility for the content of the paper.
